# Supplementary material for: Lack of GABARAP-Type Proteins Is Accompanied by Altered Golgi Morphology and Surfaceome Composition
Source: Int J Mol Sci. 2020 Dec 23;22(1):85. doi: 10.3390/ijms22010085 (PMC7795684; doi:10.3390/ijms22010085)
Supplement: Supplementary file 1 [file ijms-22-00085-s001.zip › ijms-1010577 Supporting Info.pdf]

Lack of GABARAP-type proteins is accompanied by altered Golgi morphology and surfaceome composition

Julia L. Sanwald [1,2 †], Jochen Dobner [1 †], Indra M. Simons [1,2], Gereon Poschmann [3], Kai Stühler [3,4], Alina Üffing [1,2], Silke Hoffmann [2], Dieter Willbold [1,2]

[1] Institut für Physikalische Biologie, Heinrich-Heine-Universität Düsseldorf, Universitätsstraße 1, 40225 Düsseldorf, Germany

[2] Institute of Biological Information Processing (IBI-7: Structural Biochemistry), Forschungszentrum Jülich, Leo-Brandt-Straße, 52428 Jülich, Germany

[3] Institute of Molecular Medicine I, Proteome Research, Heinrich-Heine-Universität Düsseldorf, Universitätsstraße 1, 40225 Düsseldorf, Germany; gereon.poschmann@uni-duesseldorf.de (G.P.); kai.stuehler@uni-duesseldorf.de (K.S.)

[4] Molecular Proteomics Laboratory, Biologisch-Medizinisches Forschungszentrum (BMFZ), Heinrich-Heine-Universität Düsseldorf, Universitätsstraße 1, 40225 Düsseldorf, Germany

[†] Authors contributed equally to this work

Supplementary Methods are related to Figures S1, S4

Supplementary Figure S1 is related to Figure 1

Supplementary Figure S2 is related to Figure 1

Supplementary Figure S3 is related to Figure 1

Supplementary Figure S4 is related to Figure 3

Supplementary Figure S5 is related to Figure S4

Supplementary Table S1 is related to Figure 3B

Supplementary Methods

Live-cell imaging with fluorescently labelled ceramide

HEK293 Flp-In T-REx cells (3 x 10^5) were seeded into fibronectin coated 35 mm imaging dishes (Cat. No. 81158, ibidi) and cultured for 24 h in DMEM supplemented with 10 % FCS. Staining of the cells (WT and as GABARAP(s) SKO, DKO, or TKO) with BODIPY-FL C5-ceramide (Cat. No. B-22650, Life Technologies) was conducted according to the manufacturer's instructions. Briefly, cells were rinsed in HBSS and incubated for 30 min at 4 °C with 5 µM BODIPY-FL C5-ceramide. Then, the cells were rinsed three times in ice-cold HBSS and incubated 30 min in phenol red-free DMEM supplemented with 10 % FCS. Finally, the cells were rinsed once in HBSS and stored in phenol red-free DMEM supplemented with 10 % FCS. Cells were incubated with Hoechst 33342 (Cat. No. R37605, Invitrogen, Carlsbad, CA, USA) according to the manufacturer's instructions for nuclei staining. BODIPY-FL C5-ceramide was visualised by LSM using the 488 nm channel (MBS 488).

Fluorescence-activated cell sorting

Surface levels of HLA-A were analysed under steady-state conditions in HEK293 WT and GABARAP/L1/L2^{TKO} cells. In brief, 1 * 10^6 cells of 90 % confluent cells were washed twice with ice-cold PBS and resuspended in 100 µl ice-cold FACS buffer (2 mM EGTA, 1 %FBS, 25 mM HEPES in PBS) containing 1:20 diluted HLA-A3 antibody conjugated to PE (ThermoFisher Scientific, 12-5754-42) or APC (ThermoFisher Scientific, 17-5754-42). Isotype controls (ThermoFisher Scientific, PE: Mouse IgG2a K, 12-4724-81, APC: Mouse IgG2a K, 17-4724-81) After 30 min incubation on ice in the dark, cells were washed thrice with ice-cold FACS buffer, resuspended in 0.5 ml FACS buffer and analysed by flow cytometry (Aria III, BD Bioscience, Franklin Lakes, USA). Cells were gated according to their size (SSC-A x FSC-A) and being single cells (FSC-A x FSC-H). Median fluorescence intensity of GABARAP/L1/L2^{TKO} cells was calculated relative to HEK293 WT intensity. Statistical significance was inferred as calculated by Welch's t-test using GraphPad Prism (version 8).

Immunoblotting

For proof-of-method immunoblotting, 10 µg of surface-enriched proteins were diluted with 4 x Lämmli's buffer (250 mM Tris-HCl pH 6.8, 40 % glycerol, 5 % SDS, 0.005 % bromophenol blue) containing 8 % fresh 2-mercaptoethanol. After samples were boiled for 5 min at 95 °C, surface fraction lysates were applied on 8 % SDS-PAA gel. After gel electrophoresis, semi-dry blotting of proteins onto 0.4 µm µm polyvinyliden fluoride membrane was performed for 1 h 30 min at 77 mA constant current. Unspecific binding sites were blocked for 1 h at RT with 5 % BSA in TBS-T (TBS, 0.1 % Tween-20) and membrane incubated with primary antibody for TFRC (#13208, Cell Signaling Technologies, Danvers, USA) at 1:1000 dilution overnight at 4 °C. After washing (three times with TBS-T) and incubation with 1:5000 diluted fluorescently labelled secondary antibody (ab150083, abcam) for 1 h at RT, target protein was

visualised using BioRad Imager. Statistical significance was inferred as calculated by Student's t-test using GraphPad Prism (version 8).

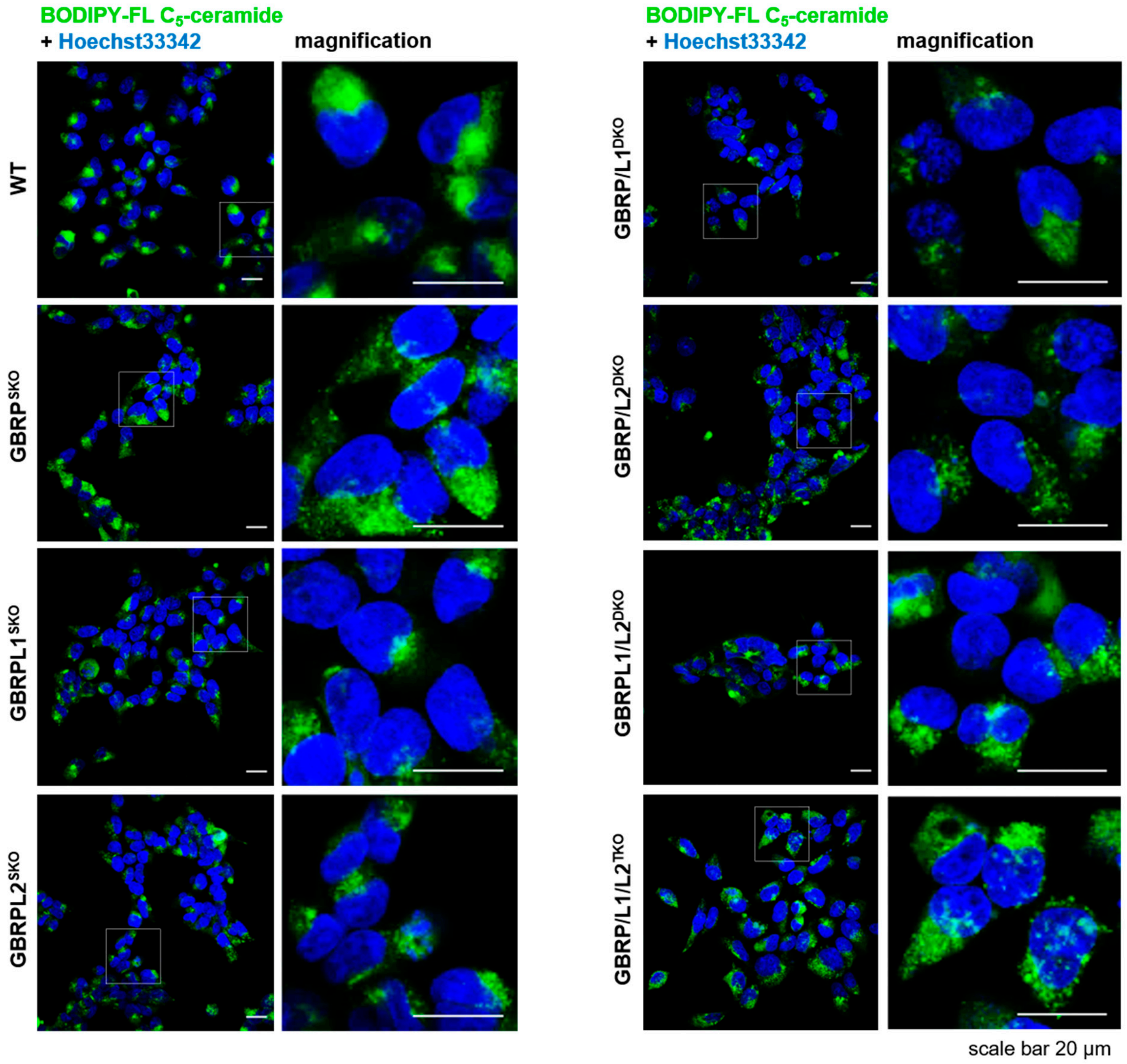

Supplementary Figure S1. Loss of GABARAP leads to a redistribution of Golgi-localised ceramide. HEK^{WT} (WT) or HEK cells with deficiency for one (SKO), two (DKO), or all three (TKO) of the GABARAPs (GBRPs) were cultured for 24 h at 37 °C and 5 % CO_2 in fibronectin coated 35 mm imaging dishes, stained with BODIPY-FL C_5-ceramide according to the manufacturer's instructions (Cat. No. B-22650, Life Technologies), and visualised by confocal fluorescence microscopy. Nuclei were counterstained with Hoechst 33342. Cells were recorded as z-stacks and the slices of each stack were combined in ImageJ by applying the function „SUMSLICES". For each condition, a representative image of five frames is shown. Scale bar, 20 µm.

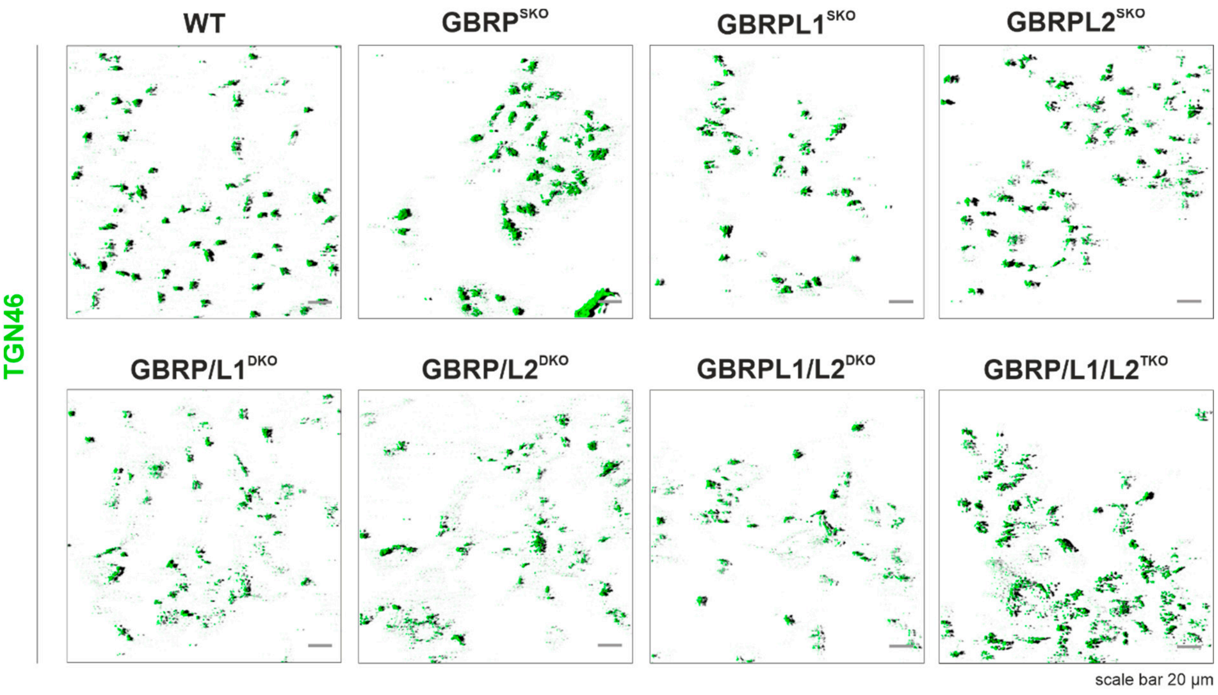

Supplementary Figure S2. 3D visualisation of the *trans*-Golgi morphology in HEK293 WT cells and under various GABARAP-type protein deficiencies. Individual planes of each stack were displayed as 3D image in ZEN 2.3 SP1 FP1 (black edition). The reconstructions relate to the respective images in Figure 1A. Scale bar, 20 µm.

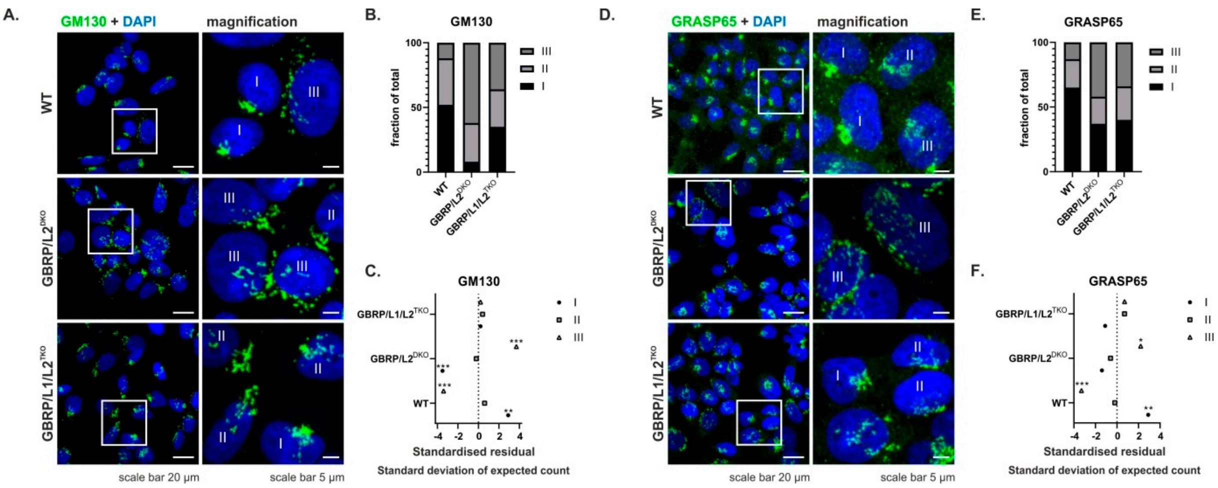

Supplementary Figure S3. Influence of GABARAP-type protein deficiency on *cis*-Golgi morphology. (A) WT, GABARAP/L2^{DKO} or GABARAP/L1/L2^{TKO} cells were fixed (4 % PFA), immunolabelled with anti-human GM130 antibody, and visualised by confocal fluorescence microscopy. Nuclei were counterstained with DAPI. Scale bar total = 20 µm, scale bar zoom-in = 5 µm. (B) Distribution of percentage of cells per cell type assigned to Golgi structure category I (compact), II (partly compact), and III (dispersed) according to GM130 staining. (C) Standardised residual distribution of analysed genotypes. Per cell type, in total ≥ 63 cells from three individual experiments were analysed and categorised by visual judgement. Asterisks indicate significant differences from the mean based on the standardised residual distribution with: $|z| \geq 2.58$ ** ($p \leq 0.01$), $|z| \geq 3.29$ *** ($p \leq 0.001$). (D) WT, GABARAP/L2^{DKO} or GABARAP/L1/L2^{TKO} cells were fixed (4 % PFA), immunolabelled with anti-human GRASP65 antibody, and visualised by confocal fluorescence microscopy. Nuclei were counterstained with DAPI. Scale bar total = 20 µm, scale bar zoom-in = 5 µm. (B) Distribution of percentage of cells per cell type assigned to Golgi structure category I (compact), II (partly compact), and III (dispersed) according to GRASP65 staining. (C) Standardised residual distribution of analysed genotypes. Per cell type, in total ≥ 103 cells were analysed and categorised by visual judgement. Asterisks indicate

significant differences from the mean based on the standardised residual distribution with: |z| ≥ 1.96 * ($p \leq 0.05$), |z| ≥ 2.58 ** ($p \leq 0.01$), |z| ≥ 3.29 *** ($p \leq 0.001$).

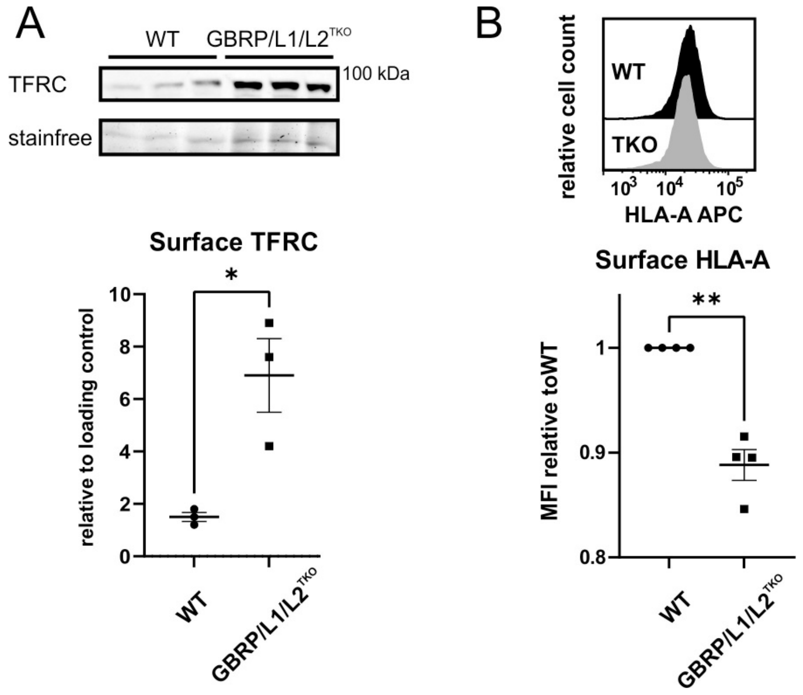

Supplementary Figure S4. Validation of surface proteome mass-spectrometry analysis by two independent methods. (A) Cell surface protein-enriched fractions were analysed by immunoblot. Surface levels of TFRC are shown for three independent experiments. Asterisk marks significant differences between GABARAP/L1/L2^{TKO} and WT cells as calculated using independent t-test. $p \leq 0.05$ = *. (B) Surface levels of MHC-I subtype HLA-A were analysed by fluorescence-activated cell sorting. Representative histograms of four independent experiments of single cells stained with HLA-A antibody are shown. Significant differences of median fluorescence intensities (MFI) between WT and GABARAP/L1/L2^{TKO} cells are marked by asterisk as calculated by Welch's t-test. $p \leq 0.01$ = **. GBRP = GABARAP. APC = Allophycocyanin.

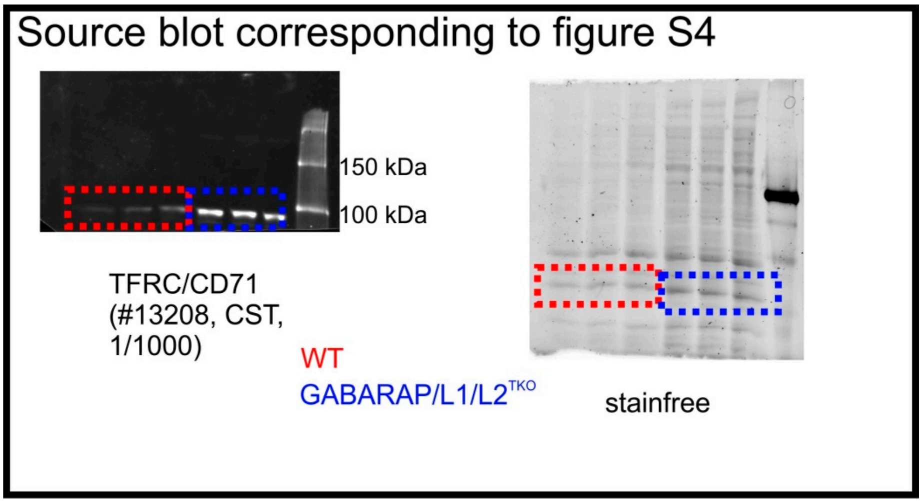

Supplementary Figure S5. Source blot corresponding to Figure S4. Regions used for display and analysis are indicated.

Supplementary Table S1. Raw data of mass-spectrometry analysis of surface-enriched proteomes of HEK293 WT and GABARAP/L1/L2^{TKO} cells can be found in the attached excel file Table S1 Mass-spectrometry data set.

Supplementary Table S2. Significantly different expressed surface-annotated proteins. For consistency, proteins are sorted as depicted in Figure 3B. Respective raw data are given in Supplementary Table S1.

GENE SYMBOL	GENE NAME	GENE ID	UNIPROT	CSPA CONFIDENCE LEVEL	DIFFERENCE TKO-WT
**PODXL2**	podocalyxin like 2	50512	Q9NZ53	1	1.95089
**CNNM4**	cyclin and CBS domain divalent metal cation transport mediator 4	26504	Q6P4Q7	1	0.801802
**ITGA7**	integrin subunit alpha 7	3679	Q13683	1	0.516933
**ADAM15**	ADAM metallopeptidase domain 15	8751	Q13444	1	0.575086
**KIRREL**	Kin of IRRE-like protein 1	55243	Q96J84	1	0.514684
**CD59**	CD59 molecule (CD59 blood group)	966	P13987	1	0.559599
**ITPRIP**	inositol 1,4,5-trisphosphate receptor interacting protein	85450	Q8IWB1	1	0.417335
**GGCX**	gamma-glutamyl carboxylase	2677	P38435	2	0.703851
**ECE1**	endothelin converting enzyme 1	1889	P42892	1	1.0075
**CNNM2**	cyclin and CBS domain divalent metal cation transport mediator 2	54805	Q9H8M5	1	0.859415
**INSR**	Insulin receptor	3643	P06213	1	0.912155
**SMPDL3B**	sphingomyelin phosphodiesterase acid like 3B	27293	Q92485	1	1.01647
**HNRNPK**	heterogeneous nuclear ribonucleoprotein K	3190	P61978	1	0.694312
**SLC4A7**	solute carrier family 4 member 7	9497	Q9Y6M7	1	0.592821
**TPBG**	trophoblast glycoprotein	7162	Q13641	1	0.787366
**NCSTN**	nicastrin	23385	Q92542	1	0.772031
**M6PR**	mannose-6-phosphate receptor, cation dependent	4074	P20645	1	0.557621
**TFRC**	transferrin receptor	7037	P02786	1	0.422732
**PTPRF**	protein tyrosine phosphatase receptor type F	5792	P10586	1	0.287566
**TMED7**	transmembrane p24 trafficking protein 7	51014	Q9Y3B3	1	0.367156
**ASIC1**	acid sensing ion channel subunit 1	41	P78348	1	0.541489
**SEL1L**	SEL1L adaptor subunit of ERAD E3 ubiquitin ligase	6400	Q9UBV2	1	0.604953
**ABCC1**	ATP binding cassette subfamily C member 1	4363	P33527	1	0.578134
**ANO6**	anoctamin 6	196527	Q4KMQ2	1	0.478563
**TYRO3**	TYRO3 protein tyrosine kinase	7301	Q06418	1	0.544295
**SLC39A14**	solute carrier family 39 member 14	23516	Q15043	1	0.594535
**NPTN**	neuroplastin	27020	Q9Y639	1	0.499769

**ALCAM**	activated leukocyte cell adhesion molecule	214	Q13740	1	0.458814
**ATP1A1**	ATPase Na+/K+ transporting subunit alpha 1	476	P05023	1	0.340416
**NCR3LG1**	natural killer cell cytotoxicity receptor 3 ligand 1	374383	Q68D85	1	0.578246
**ATP1B3**	ATPase Na+/K+ transporting subunit beta3	483	P54709	1	0.570178
**SLC3A2**	solute carrier family 3 member 2	6520	P08195	1	0.643518
**ACTN1**	actinin alpha 1	87	P12814	1	0.712156
**JAM3**	junctional adhesion molecule 3	83700	Q9BX67	1	0.468754
**CD276**	CD276 molecule	80381	Q5ZPR3	1	0.428201
**CADM1**	cell adhesion molecule 1	23705	Q9BY67	1	0.497293
**MIA3**	MIA SH3 domain ER export factor 3	375056	5JRA6	1	-0.909785
**HLA-C**	major histocompatibility complex, class I, C	3107	P10321	1	-0.539395
**HLA-A**	major histocompatibility complex, class I, A	3105	P04439	1	-1.0196
**LGALS3BP**	galectin 3 binding protein	3959	Q08380	1	-0.991107
**CLPTM1L**	cleft palate transmembrane protein 1-like	81037	Q96KA5	2	-0.741051
**MFAP3**	microfibril associated protein 3	4238	P55082	1	-0.768297
**TOR1AIP1**	torsin 1A interacting protein 1	26092	Q5JTV8	2	-0.685934
**RPN1**	ribophorin 1	6184	P04843	1	-0.358063
**TMEM259**	transmembrane protein 259	91304	Q4ZIN3	2	-0.314032
**STT3A**	STT3 oligosaccharyltransferase complex catalytic subunit A	3703	P46977	2	-0.59739
**CACHD1**	cache domain containing 1	57685	Q5VU97	1	-0.580828
**CNTN1**	contactin 1	1272	Q12860	1	-0.711767
**EMC1**	ER membrane protein complex subunit 1	23065	Q8N766	1	-0.646965
**MCAM**	melanoma cell adhesion molecule	4162	P43121	1	-0.682648
**F11R**	F11 receptor	50848	Q9Y624	1	-0.478582
**ITGA4**	integrin subunit alpha 4	3676	P13612	1	-0.446824
**EPHA4**	EPH receptor A4	2043	P54764	1	-0.57585
**EFNB1**	ephrin B1	1947	P98172	1	-0.896389
**EPCAM**	epithelial cell adhesion molecule	4072	P16422	1	-0.78135
**BRI3BP**	BRI3 binding protein	140707	Q8WY22	2	-0.665538
**CD46**	CD46 molecule	4179	P15529	1	-0.848529
**CAPNS1**	calpain small subunit 1	826	P04632	1	-0.562122
